# Effect of Fatigue on Word Production in Aphasia

**Published:** 2024

**Authors:** Daniel Mirman, Anna Krason, Malathi Thothathiri, Erica L. Middleton

**Affiliations:** Department of Psychology, University of Edinburgh, 7 George Square, Edinburgh, EH8 9JZ, UK; Moss Rehabilitation Research Institute, 50 Township Line Road, Elkins Park, PA 19027, USA; Department of Speech, Language & Hearing Sciences, George Washington University, 2115 G Street NW, Washington DC 20052, USA; Moss Rehabilitation Research Institute, 50 Township Line Road, Elkins Park, PA 19027, USA

**Keywords:** effort, fatigue, word production, aphasia

## Abstract

Speech production in aphasia is often described as “effortful”, though the consequences of consistent, high degrees of cognitive effort have not been explored. Using recent work on mental effort as a theoretical framework, the present study examined how effort-related fatigue produces decrements in performance in picture naming among participants with post-stroke aphasia. We analyzed three data sets from prior studies where participants completed a large picture naming test. Decreasing naming accuracy across trials was statistically significant in two of the three samples. There were also significant effects of practice (better performance on a second test administration), word frequency (better performance for more frequent words), and word length (better performance for shorter words). These results are the first concrete demonstration of fatigue affecting performance on a language task in post-stroke aphasia. They open a new avenue for research on mental effort/fatigue with potential implications for aphasia assessment, treatment, and management.

## Introduction

Recent work on mental effort has developed a rational economic model where effort and/or cognitive control are considered finite resources and allocation of those resources is based on a rational calculation of expected rewards (e.g., Kool & Botvinick, 2018; Shenhav et al., 2017). This approach works well in contexts where the rewards can be easily quantified, such as economic decisions. However, in many tasks – such as language production – the rewards are harder to quantify. For such contexts, a somewhat different framework, based on physical effort, may be more useful. Physical effort can be measured by defining maximum effort in concrete terms (e.g., heaviest weight that a participant can lift) and lower effort, as well as fatigue, can be expressed as proportions of that maximum (Steele, 2020, 2023). This can be extended to mental effort by using Item Response Theory to quantify task difficulty and defining maximum effort for a given task (e.g., n-back) as the most difficult version that a participant is able to perform accurately. Then effort and fatigue can be expressed as performance relative to that maximum.

These theoretical frameworks have the potential to shed light on post-stroke fatigue, a frequent and often severe symptom that continues into the chronic stage years after stroke (e.g., English et al., 2023; Glader et al., 2002; Markus, 2023), with profound effects on quality of life. Approximately half of stroke survivors experience post-stroke fatigue and while other symptoms typically decrease, the prevalence of post-stroke fatigue does not decrease over time after stroke (Zhan et al., 2023). Stroke survivors rate understanding and reducing fatigue among the highest research priorities (Hill et al., 2022). The neural or physiological basis of post-stroke fatigue remains unknown. It may be that the post-stroke fatigue arises (at least partly) from increased exertion of cognitive/mental effort. That is, fatigue may result from stroke survivors having to consistently exert high degrees of cognitive/mental effort in order to carry out routine tasks. To our knowledge, there have not been prior attempts to apply theories or methods of research on cognitive effort to the problem of post-stroke fatigue.

A good starting point for this integration is speech production in aphasia, which is often described as “effortful”. Compared to neurologically intact participants, individuals with aphasia report higher levels of perceived effort during speech production tasks (e.g., Harmon et al., 2019) and speech-language pathologists report observing fatigue during aphasia therapy sessions, with resulting decreases in performance (e.g., Riley, 2017).

Putting this in terms of the physical effort framework, for neurologically intact participants, their ability to produce speech far exceeds the difficulty of the task, so (in general) only a small amount of effort is required. For individuals with aphasia, their ability to produce speech has been substantially reduced by brain injury, so the same task is now much closer to their maximum and therefore requires much more effort. Continually exerting this high level of effort produces fatigue (i.e., a reduction in the available cognitive resources), which leads to worse performance on the task.

In the present study, we consider confrontation naming: when presented with the picture of a familiar object, saying its name. This task is widely used in the clinic to examine problems with retrieving words in the course of production (i.e., naming impairment) in people with aphasia. Naming impairment is a ubiquitous feature of aphasia, is a major impediment to communication, and thus is a common target of speech-language therapy. Here we used data from a large-scale picture naming task as a controlled test and first step toward understanding the relationship between cognitive effort, fatigue, and language performance in post-stroke aphasia.

## Methods

### Data

The data were collected by Middleton and colleagues from three of their prior studies that all included a large picture naming test of common, everyday objects. All participants were native English speakers with chronic aphasia (>6 months post onset) secondary to stroke and had some degree of measurable naming impairment. In each study, a large-scale picture naming test was administered in sessions involving presentation of at least 300 items. The full test was administered a second time, in a different week. Item order was randomized per administration per participant. On each naming trial, the object was displayed, the participant attempted to name the object, and no feedback of any kind was provided.

Sample 1: 17 participants who completed a 660-item picture naming test in two 330-item sessions, which was administered a second time in a different week (Tian et al., 2023).

Sample 2: 23 participants who completed the same 660-item picture naming test in two 330-item sessions, which was administered a second time in a different week (Middleton et al., 2022).

Sample 3: 10 participants who completed a 300-item picture naming test in a single session, which was administered a second time in a different week (Patra et al., 2023).

Samples 1 and 2 were not selected based on aphasia subtype or severity; Sample 3 was intended to study lexical retrieval difficulty, so those participants were selected to have word production impairment in the context of intact semantics and minimal repetition deficits. Some participants completed multiple studies, so across the three samples there were 55,388 analyzable observations in 50 data sets from 36 unique participants.

### Analysis

The same analysis approach was applied to each sample. The samples were analyzed separately because there were minor differences in the administration procedure (e.g., time allowed to make a response) and scoring procedure (i.e., leniency about what was considered a correct response). A logistic mixed-effects regression model was used to analyze trial-level accuracy (correct vs incorrect response) with critical fixed effects of trial number, test number (centered), and their interaction. The fixed effects also included basic controls for item difficulty: word frequency (log10 frequency per million words, centered) and word length (in phonemes, centered). The model random effects were by-participant random intercepts and random slopes of trial number, and by-item random intercepts. The model was implemented using the lme4 package version 1.1–34 (Bates et al., 2015) in R version 4.3.1:

glmer(Correct ~ Trial_Num * Test_Num +
          Frequency + Length +
     (1 + Trial_Num | ParticipantID) +
     (1 | Item_Num), family=binomial, …)


## Results

### Sample 1 (N=17)

**Figure 1.**
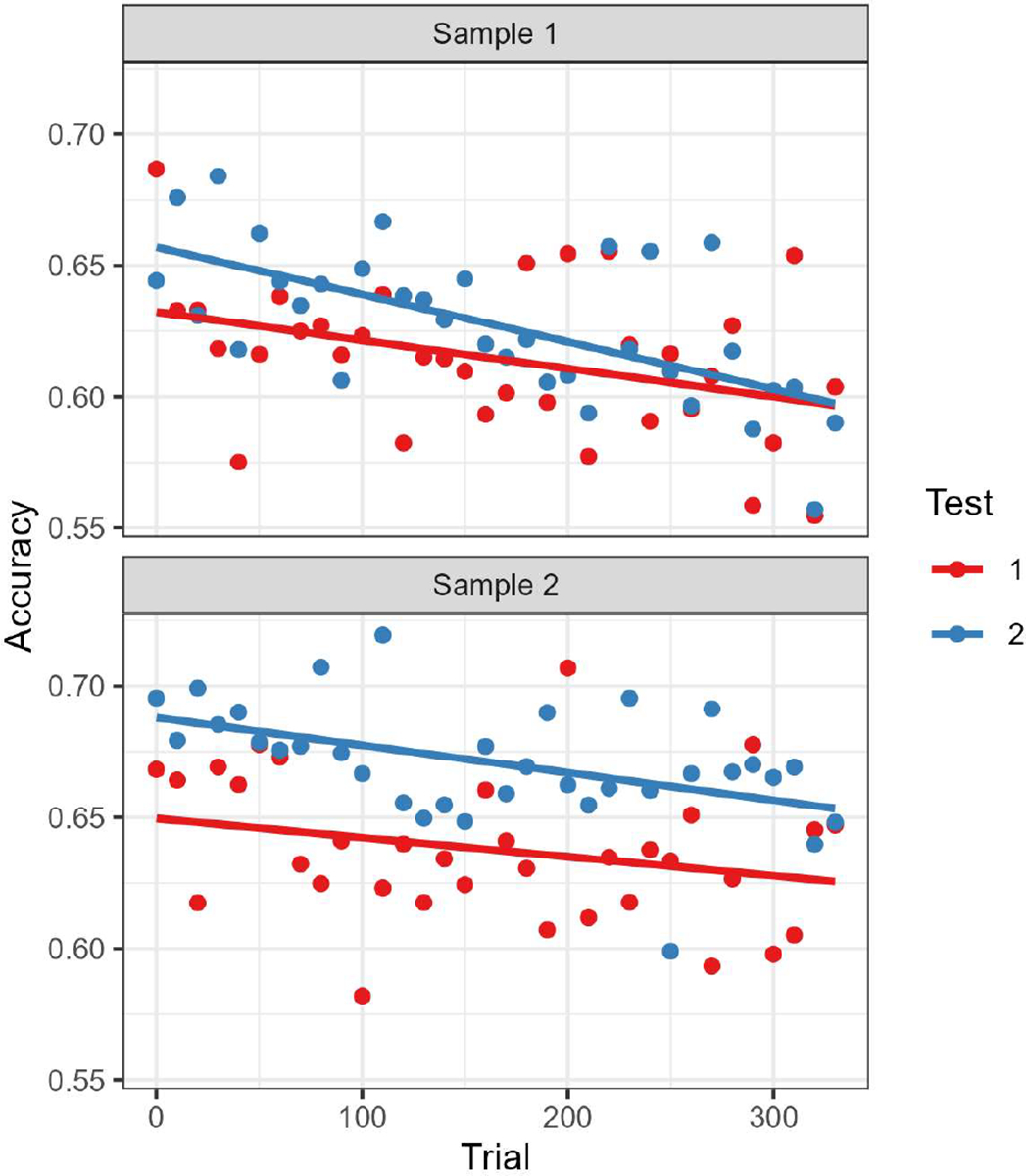
Accuracy as a function of trial number and test number in samples 1 (top) and 2 (bottom). Points indicate average accuracy across participants for bins of 10 trials. Results from the first test administration are shown in red, from the second test administration in blue. The lines are best fit trendlines. The downward slope of the lines reflects the fatigue effect (lower accuracy later in the testing session), the blue line being higher reflects the practice effect (better performance on the second test administration than the first).

Results of the analysis of sample 1 are shown in Table 1 and the data are plotted in the top panel of Figure 1. There was a significant negative effect of trial number (Estimate = −0.76, SE = 0.18, p < 0.001) indicating that naming performance decreased across trials. There was also a significant effect of test number (Estimate = −0.08, SE = 0.03, p = 0.022), reflecting higher accuracy on the second administration than the first, but no statistically significant interaction between test and trial number (i.e., practice and fatigue effects did not interact). Not surprisingly, there were strong effects of word frequency (higher accuracy for more frequent words) and word length (lower accuracy for longer words).

### Sample 2 (N=23)

Results of the analysis of sample 2 were very similar to results for sample 1. They are shown in Table 2 and the data are plotted in the bottom panel of Figure 1. There was a significant negative effect of trial number (Estimate = −0.50, SE = 0.15, p < 0.001) indicating that naming performance decreased across trials. There was also a significant effect of test number (Estimate = −0.13, SE = 0.03, p = 0.001), reflecting higher accuracy on the second administration than the first, but no interaction between test and trial number (i.e., practice and fatigue effects did not interact), and strong effects of word frequency (higher accuracy for more frequent words) and word length (lower accuracy for longer words).

### Sample 3 (N=10)

Results of the analysis of sample 3 are shown in Table 3. The effect of trial number was in the same direction as for samples 1 and 2, but was smaller and not statistically significant (Estimate = −0.28, SE = 0.38, p = 0.46). The effect of test number was also not significant, and there was no significant interaction between trial and test number. That is, for sample 3 there was not a reliable decrease in performance across trials, nor an effect of practice or interaction between practice and fatigue. However, as in samples 1 and 2, there were strong effects of item difficulty: a positive effect of word frequency (higher accuracy for more frequent words) and a negative effect of word length (lower accuracy for longer words).

### Individual Differences

We conducted follow-up analyses as a first step toward assessing whether variability in these fatigue effects reflects individual differences in language impairment. That is, whether the slope of an individual participant’s decline in naming performance is associated with performance on other language tests. Individual participant fatigue effects were quantified using the random effect estimates from the models (i.e., the by-participant random slopes of trial number), which are estimated as coming from a normal distribution with a mean of 0. That is, a random slope estimate of 0 corresponds to the overall group mean, a negative slope estimate indicates a faster-than-average decline in naming performance, and a positive slope estimate indicates slower-than-average decline.

These estimates were extracted from each sample’s model and transformed into z-scores (each sample had different slope variance estimates so this z-score transformation put them on the same scale). These fatigue effect estimates were combined with scores from a battery of cognitive and language tests that the participants had completed separately from the naming studies. Five scores were selected for these analyses:

- Western Aphasia Battery Aphasia Quotient (WAB AQ) (Kertesz, 1982): an overall assessment of aphasia severity.
- Philadelphia Naming Test (PNT) (Roach et al., 1996): an independent picture naming test consisting of 175 items.
- Philadelphia Repetition Test (PRT) (Roach et al., 1996): a word repetition test using the same items as the PNT.
- Camel and Cactus Test (CCT) (Bozeat et al., 2000): a picture matching test of semantic cognition that does not require verbal comprehension or production.
- Synonym Triplets (SYN) (Martin et al., 2006): a synonym judgment task to test verbal semantic knowledge.

These scores were selected as an initial assessment of how fatigue effects are related to general aphasia severity (WAB AQ), picture naming ability (PNT), and the two primary stages of word production: semantic cognition (non-verbal, CCT, and verbal, SYN) and output phonology (PRT).

Linear regressions were used to test whether the slope estimates were predicted by each test score and its interaction with study (i.e., in case these differed between samples). As summarized in Table 4, none of the language impairment scores were statistically significantly associated with these fatigue effect estimates and this did not differ between samples.

## Discussion

### Summary of Results

Across two samples (Samples 1 and 2), with a total N=40, there was a very consistent pattern. Participants with aphasia showed declining picture naming performance over the course of a session of 330 picture naming trials, consistent with an effect of cognitive/mental fatigue. In addition, they showed a practice effect - higher accuracy on a second test (on a different week) of the same items - and no interaction between the fatigue effect and the practice effect. Trial order was randomized for each testing session and we additionally accounted for item difficulty by including word frequency and length in the models, so the decreasing performance over time is unlikely to be caused by consistent differences between items presented late vs early in the testing session.

A third sample of 10 participants exhibited the expected word frequency and length effects (higher accuracy for frequent words and lower accuracy for longer words) as for the first and second samples. But we did not find fatigue or practice effects. This sample had about half the number of participants and the sessions were 10% shorter, so the estimates of fatigue effects are less precise. However, the estimates themselves were numerically smaller than in the other samples, so statistical power is unlikely to be the only factor. A possibly more important factor is that this sample was more homogeneous than the first two samples: the third sample was restricted to participants with word production impairment but intact semantics and minimal repetition deficits. It may be that fatigue effects are larger for individuals outside of this more narrow set of inclusion criteria.

Follow-up analyses of individual differences did not reveal any associations between magnitude of fatigue effects and severity of language impairment (WAB AQ), severity of picture naming impairment (PNT), output phonology (PRT), or semantic processing (CCT, synonym triplets).

### Implications

To our knowledge, this is the first concrete demonstration of fatigue affecting performance on a language task in post-stroke aphasia. As such, it forms a potential bridge between basic research on mental effort and translational research on post-stroke fatigue. Post-stroke fatigue is pervasive and debilitating, with higher prevalence than would be expected just based on age and disability. Fatigue is, of course, a normal response to exertion, but for neurologically intact individuals this is a predictable and transient experience, whereas individuals with neurological conditions report chronic fatigue that is qualitatively different from their experience prior to the illness (Kluger et al., 2013). Stroke survivors rate understanding and reducing fatigue among the highest research priorities.

Efforts to determine the neural or physiological basis of post-stroke fatigue have not (yet) produced clear results. Cognitive science offers a different perspective: post-stroke fatigue may result (at least partly) from stroke survivors having to consistently exert high degrees of cognitive/mental effort in order to carry out routine tasks. In the current study, the fatigue would build up as individuals with aphasia exert high degrees of cognitive/mental effort in order to name pictures of familiar objects (a very easy task for neurologically intact individuals). Further research may help to understand how the overall post-stroke fatigue phenomenon emerges from these fine-grained expenditures of cognitive/mental effort.

Consideration of fatigue may also have implications for clinical neuropsychological assessment and experimental measurement of deficits in the lab. Specifically, performance can be influenced by fatigue in addition to underlying cognitive deficits. Similarly, this may have implications for structuring treatment schedules. Considering an individual’s particular profile of effort, task tolerance, and fatigue may be important for optimising the schedule of treatment. But that requires an effective way to concretely measure effort and fatigue – a contribution that could come from developing and applying cognitive theories of mental effort.

### Further Considerations

For the present analyses, only data from participants with post-stroke aphasia were available, so it was not possible to compare with a neurologically intact control group. Effects of cognitive fatigue are generally common, including in neurologically healthy young adults performing standard experimental psychology tasks (e.g., Baayen & Milin, 2010; Faber et al., 2012), so it is possible that neurologically intact participants would exhibit the same fatigue effects observed in this study. However, there are two strong reasons to expect fatigue effects to be larger for individuals with aphasia. First, word production is substantially more effortful for individuals with aphasia; the greater expenditure of cognitive effort should produce larger fatigue effects. Second, neurological damage may increase fatigability because of reduced neural resources or related physiological changes (e.g., changes in energy regulation). As a result, even if the individual trials were no more difficult for them, stroke survivors could fatigue more quickly than neurologically intact participants. Focused study designs with different neurological groups would be needed to investigate these mechanisms.

Future research on mental effort and fatigue will also need to distinguish between general fatigue and task-specific fatigue. This has implications for cognitive theories of mental effort (to what extent is it a general resource vs task-specific allocation) and translational implications for optimising assessment and treatment protocols (e.g., can mixing tasks mitigate effects of fatigue). These studies should also consider the effects of frustration and, in some cases, emotional strain that arise from working on a difficult task. For individuals with aphasia, in real-world contexts, the response of the communication partner will also have substantial impact (Harmon, 2020).

Although the preliminary analyses of individual differences in this study did not reveal any statistically significant effects, this deserves further study. First, it may be that we did not quantify individual participant fatigue effects in the optimal way, other strategies are available and can be explored. Second, we only considered closely related cognitive-neuropsychological differences, but other differences, such as personality (e.g., frustration tolerance) and neurological (lesion size and location) factors should be explored.

Finally, we examined fatigue in the context of a laboratory/clinical task: picture naming. This task does have some ecological validity (word retrieval is an important aspect of communication). We see this as a step toward bridging the substantial gap between laboratory cognitive research on mental effort and clinical research on post-stroke fatigue. However, the clinical impact would be strengthened by an understanding of the effects of cognitive/mental fatigue on functional communication and quality of life.

## Figures and Tables

**Table 1:** Results for Sample 1.

Fixed Effect	Est (SE)	Odds Ratio (CI)	p-value
Trial Number	−0.76 (0.18)	0.47 (0.33−0.66)	<0.001
Test Number	−0.08 (0.03)	0.93 (0.87−0.99)	0.022
Trial × Test	0.22 (0.17)	1.25 (0.89−1.75)	0.20
Word Frequency	0.88 (0.07)	2.40 (2.11−2.74)	<0.001
Word Length	−0.90 (0.20)	0.41 (0.27−0.61)	<0.001

**Table 2:** Results for Sample 2.

Fixed Effect	Est (SE)	Odds Ratio (CI)	p-value
Trial Number	−0.50 (0.15)	0.61 (0.45−0.82)	<0.001
Test Number	−0.13 (0.03)	0.88 (0.83−0.93)	0.001
Trial × Test	0.17 (0.15)	1.19 (0.88−1.59)	0.26
Word Frequency	0.83 (0.07)	2.29 (2.01−2.61)	<0.001
Word Length	−2.08 (0.20)	0.12 (0.08−0.19)	<0.001

**Table 3:** Results for Sample 3.

Fixed Effect	Est (SE)	Odds Ratio (CI)	p-value
Trial Number	−0.28 (0.38)	0.75 (0.35−1.60)	0.46
Test Number	0.04 (0.07)	1.04 (0.91−1.19)	0.56
Trial × Test	−0.49 (0.39)	0.61 (0.29−1.30)	0.26
Word Frequency	0.75 (0.15)	2.12 (1.59−2.82)	<0.001
Word Length	−1.77 (0.41)	0.17 (0.08−0.38)	<0.001

**Table 4:** Results of Individual Difference Analyses.

Predictor	Predictor (F(1,44))	Predictor × Sample (F(2,44))
WAB AQ	F=0.20, p > 0.6	F=2.23, p > 0.3
PNT	F=0.31, p > 0.5	F=1.29, p > 0.5
PRT	F=0.07, p > 0.7	F=0.09, p > 0.9
CCT	F=0.26, p > 0.6	F=0.84, p > 0.4
SYN	F=0.26, p > 0.6	F=0.15, p > 0.8
